# Effect of Melatonin for Regulating Mesenchymal Stromal Cells and Derived Extracellular Vesicles

**DOI:** 10.3389/fcell.2021.717913

**Published:** 2021-09-01

**Authors:** Zi-Yi Feng, Shu-De Yang, Ting Wang, Shu Guo

**Affiliations:** Department of Plastic Surgery, The First Affiliated Hospital of China Medical University, Shenyang, China

**Keywords:** melatonin, mesenchymal stromal cells, extracellular vesicles, regenerative medicine, biological therapeutics, paracrine effect

## Abstract

Melatonin is a hormone, synthesized in the pineal gland, which primarily controls the circadian rhythm of the body. In recent years, melatonin has also been shown to regulate metabolism, provide neuroprotection, and act as an anti-inflammatory, free radical scavenger. There has also been a recent research interest in the role of melatonin in regulating mesenchymal stromal cells (MSCs). MSCs are pivotal for their ability to differentiate into a variety of different tissues. There is also increasing evidence for the therapeutic prospects of MSCs *via* paracrine signaling. In addition to secreting cytokines and chemokines, MSCs can secrete extracellular vesicles (EVs), allowing them to respond to injury and promote tissue regeneration. While there has been a major research interest in the use of MSCs for regenerative medicine, the clinical application is limited by many risks, including tumorigenicity, senescence, and sensitivity to toxic environments. The use of MSC-derived EVs for cell-free therapy can potentially avoid the disadvantages of MSCs, which makes this an exciting prospect for regenerative medicine. Prior research has shown that MSCs, *via* paracrine mechanisms, can identify receptor-independent responses to melatonin and then activate a series of downstream pathways, which exert a variety of effects, including anti-tumor and anti-inflammatory effects. Here we review the synthesis of melatonin, its mechanisms of action, and the effect of melatonin on MSCs *via* paracrine signaling. Furthermore, we summarize the current clinical applications of melatonin and discuss future prospects.

## Introduction

Melatonin, a ubiquitous molecule with the chemical name *N*-acetyl-5-methoxytryptamine, was first isolated in 1958 from the pineal gland. It is locally synthesized by several different organs and tissues such as the retina, gastrointestinal tract, bone marrow, lymphocytes, skin, and the pineal gland. Decades of research have gradually elucidated its diverse functions and mechanisms of action. The primary function of melatonin is regulation of dark signals, and this may also lead to regulation of circadian rhythms and seasonality. The circadian rhythm refers to the alterations of life activities within a roughly 24-h cycle. There is clear evidence that circadian rhythms are closely related to physiological function, learning and memory ability, emotional stability, and work efficiency. Circadian rhythm sleep disorders are caused by changes in the central circadian rhythm systromal or imbalances between endogenous circadian rhythms and external environments (Zhu and Zee, 2012). While other hypnotics induce sleep by changing the sleep patterns and result in undesirable side effects, exogenous melatonin acutely induces and maintains sleep with few side effects (Cajochen et al., 1996). Melatonin and the antidepressant agomelatine have the same mechanism of action, and there is evidence that adding adjuvant melatonin to anti-depressant regimens may further help to control depression symptoms (Dubocovich et al., 2010; Agahi et al., 2018). Melatonin also participates in the control of energy metabolism, and reduced melatonin secretion may cause obesity (Liu et al., 2019). Research by Rahman et al. (2017) has also shown that, in combination with regular exercise, supplemental melatonin can improve insulin resistance, hypertension, and fatigue in the type 2 diabetes mellitus rat model. Melatonin is also an antioxidant and free radical scavenger, and several defensive and curative functions have been reported, including the protection of organs such as the brain and gastrointestinal tract and the prevention of cardiovascular disorders and tumors (Tordjman et al., 2017; Ashrafizadeh et al., 2021; Vaseenon et al., 2021).

Mesenchymal stromal cells (MSCs) are a type of multipotent cell that were first discovered by Friedenstein in 1968 (Friedenstein et al., 1970). They can be isolated from different tissues, including adipose tissue, muscle, bone marrow, periosteum, placenta, dental tissue, and others (Murphy et al., 2013; Spagnuolo et al., 2018). Among them, the MSCs from bone marrow, adipose tissue, and perinatal tissue utilized in clinical trials are at the same high frequency (Moll et al., 2020). MSCs are capable of self-renewal and can differentiate into multiple tissues, including bone, cartilage, adipose, tendon, and myocardium (Via et al., 2012). MSCs reside in a niche, which is a complex microenvironment, and their functions are influenced by many physical and chemical factors, peripheral cells, and hormones (Tatullo et al., 2016; Marrelli et al., 2018). In recent years, the complex paracrine mechanisms of MSCs have attracted considerable attention. Most of the therapeutic activity of MSCs can be attributed to the direct primary signals through their secretors, including a large number of cytokines, chemokines, growth factors, and subcellular vesicles (Moll et al., 2020). The extracellular vesicles (EVs) are important bioactive vesicles implicated in the paracrine effects of MSCs, and they consist of spherical bilayer lipid membranes with 50 nm to around 200 nm in diameter (Witwer et al., 2019). EVs can transfer “cargos” of proteins, DNA, lipids, cytokines and growth factors, mRNAs, and regulatory miRNAs to regulate the “fate” of recipient cells by affecting their proliferation, differentiation, migration, and gene expression. EVs are derived from the endolysosomal pathway or created by budding at endosome membranes (El Andaloussi et al., 2013; Pegtel and Gould, 2019). The most commonly used technique to isolate EVs is differential ultracentrifugation, but there are also other methods that can achieve the same aim (Kim et al., 2016). Numerous studies have demonstrated that EVs are a vital product of MSCs and resemble the effects of the parent MSCs (Wu et al., 2018). EVs are involved in intercellular transmission, cellular signal transduction, and short- or long-distance changes in cell or tissue metabolism. They can also influence tissue response to injury, infection, and disease. Thus, EVs provide a new therapeutic paradigm for cell-free MSC-based therapies. Importantly, the contents of EVs are not static; rather, they are a product of the tissue of origin, activities, and immediate intercellular neighbors of the MSC (Phinney and Pittenger, 2017).

Melatonin is one such hormone that may regulate MSC differentiation and function. In a study on rats, night-time melatonin concentrations in the bone marrow were twice as high as those in peripheral blood, and high melatonin concentrations that were still detected in the bone marrow of pinealectomized rats indicated that some melatonin can be synthesized in the bone marrow (Tan et al., 1999). What is more, the receptor-dependent and receptor-independent responses of melatonin to MSCs are supposed to happen (Luchetti et al., 2014). Recent advances have confirmed that melatonin promotes osteogenic and chondrogenic differentiation of MSCs and inhibits adipogenic differentiation (Wang et al., 2019). Melatonin has also been shown to improve the therapeutic potential of MSCs in different disease models (Hu and Li, 2019).

Herein we critically identify and evaluate current research on the functions and mechanisms of melatonin for the regulation of MSCs and their derived EVs in cell-free therapy.

## Melatonin: A Hormone Essential to Life

### Melatonin Anabolism Pathway and Receptors

**FIGURE 1:**
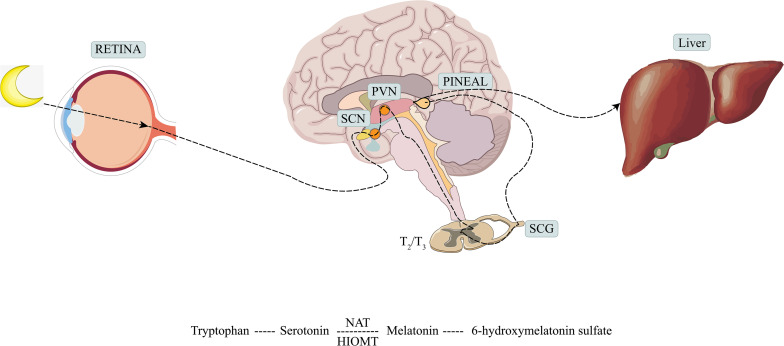
Production and metabolism of melatonin. SCN, suprachiasmatic nucleus; PVN, paraventricular nucleus; SCG, superior cervical ganglion; NAT, serotonin-*N*-acetyl transferase; HIOMT, hydroxy indole-*O*-methyl transferase.

Melatonin is a hormone synthesized mainly by the pineal gland and mediated by the retinal melanopsinergic systromal and the nervous systromal (Cipolla-Neto and Amaral, 2018), and it is produced in synchrony with the dark cycle. In this synthesis pathway, the inhibitory signals from the retina, which are especially sensitive to blue wavelengths of visible light, are lifted at night. In response, the suprachiasmatic nucleus projects directly and indirectly to the preganglionic sympathetic neurons of the first thoracic segments of the spinal cord *via* the paraventricular nucleus. Through the projection of the postganglionary sympathetic neuron of the superior cervical ganglia, it reaches the pineal gland and the synthesis process is completed (Amaral and Cipolla-Neto, 2018). The norepinephrine initiates melatonin synthesis by binding to the adrenaline receptor, and by a two-step process mediated by serotonin-*N*-acetyl transferase and hydroxy indole-*O*-methyl transferase, serotonin is then converted to melatonin (Zimmermann et al., 1993; Besharse and McMahon, 2016) (see Figure 1). Melatonin synthesis has also been reported to occur outside of the pineal gland, including in the retina, bone marrow, lymphocytes, and gastrointestinal tract of vertebrate species (Tordjman et al., 2017).

After synthesis, melatonin can be stored in the pineal gland or released into the blood or cerebrospinal fluid (CSF). Melatonin is absorbed into the blood by passive diffusion and binds only to plasma albumin. The binding rate of melatonin to plasma albumin is about 33%, but the binding is very loose and unsaturated. This may lead to a rapid entry into the organelles. Melatonin has a rather short half-life, which makes it a good candidate for intravenous injection (Deprés-Brummer et al., 1996; Hardeland et al., 2011).

Melatonin exerts its biological roles through receptor and non-receptor pathways. The non-receptor-mediated pathways refer to melatonin passage across cell membranes, which promotes the direct interactions of melatonin and other molecules based on their amphiphilic properties. Melatonin is distributed ubiquitously but unequally in the cell, where it directly detoxifies reactive oxygen and nitrogen species, stimulates antioxidant enzymes, and chelates transition metals (Reiter et al., 2016). In other cases, the mechanisms of action of melatonin are mediated by receptors. High-affinity G protein-coupled receptors MT1 (MTNR1A in humans) and MT2 (MTNR1B in humans) are found in various cellular locations, and through engagement with these receptors, melatonin can exert its effects on sleep and mood, learning and memory, and even cancer progression (Liu et al., 2016; Pistioli et al., 2021). The MT1 and MT2 receptors act as heterotrimeric Gi/Go and Gq/11 protein-coupled receptors, respectively, and can signal to downstream molecules including adenylyl cyclase, phospholipase A2, and phospholipase C. This signaling occurs by decreasing cAMP and cGMP synthesis and/or by increasing diacylglycerol and IP3 formation (Amaral and Cipolla-Neto, 2018). Recent reports have also demonstrated that melatonin can be synthesized in the mitochondrial matrix and then released to activate the mitochondrial MT1 signal transduction pathway, which inhibits stress-mediated cytochrome c release and caspase activation. This process is referred to as “automitocrine” signaling (Suofu et al., 2017). The melatonin receptors MT1 and MT2 were proven to be existent in BMSCs; this evidence suggested that melatonin may regulate the BMSCs (Dubocovich and Markowska, 2005; Slominski et al., 2012). Not just in BMSCs, the melatonin receptor antagonists, luzindole or 4P-PDOT (MT2 receptor selective), inhibited the melatonin-induced high expression of ALP activity of MSCs from other origins. The involvement of melatonin receptors especially MT2 was further proven (Radio et al., 2006; Heo et al., 2019).

The MT1 and MT2 receptors are not the only melatonin binding sites. Cytosolic enzyme quinone reductase 2 is now considered a third membrane melatonin receptor (MT3). MT3 does not initiate the classical melatonin signaling pathway, but it is involved in the detoxification of chemotherapeutic-induced cytotoxicity (Hardeland et al., 2011). Some orphan nuclear receptors, nuclear receptors without known ligands, may also act as melatonin receptors. Melatonin has been confirmed to interact with RORα (Li et al., 2020). Melatonin receptors have a variety of different probable mechanisms and the ability to regulate the different physiological, therapeutic, and pathological effects of melatonin; therefore, melatonin receptor agonists and antagonists have great therapeutic potential and should be explored further.

### Differential Time-Allocated Effects and Functions

Melatonin exerts its diverse functions through different time-allocated effects. The immediate effects of melatonin occur through the classical hormone mechanism of action, in which melatonin and molecular effectors are released into the blood or CSF. The possible functions of melatonin hormone signaling include antioxidant effects, reduction of cAMP-PKA-CREB and cGMP, increased DAG, IP3, PKC activity, and monitoring of potassium and calcium channels.

The prospective effect occurs during the day and is triggered by the disappearance of the melatonin from the night before. This effect can be subdivided into “proximal or consecutive effects” and “distal or prolonged effects,” and these separately control the super- or hypersensitization of the cAMP/PKA/CREB pathway and transcription and/or translation of the clock genes and clock-controlled genes.

The precise crosstalk between the melatonin signal and the daytime dark phase makes melatonin the primary coordinator of circadian rhythm, and this function is often considered a chronobiotic effect. Meanwhile, the relationship of melatonin with seasonal change is referred to as the seasonal effect. Notably, maternal melatonin regulates the behavior and physiology of the child in response to the environmental light/dark cycle after birth, and this is known as the transgenerational effect (Amaral and Cipolla-Neto, 2018; Cipolla-Neto and Amaral, 2018).

## Mesenchymal Stromal Cells and their Derived Extracellular Vesicles

### Extracellular Vesicles Are Representative of Paracrine Effects

Mesenchymal stromal cells are pivotal for their ability to differentiate into a variety of cell and tissue types in regenerative medicine. In recent years, research on MSCs has become a hot topic, particularly due to their wide distribution in the body and great proliferative potential (Murphy et al., 2013). In addition to their capacity to differentiate into a variety of mesenchymal and non-mesenchymal cell lineages and their tissue repair capabilities, MSCs also exert paracrine functions that may have useful therapeutic prospects (Pankajakshan and Agrawal, 2014). It has been shown that the paracrine effects of MSCs are responsible for most of their effects on the body and that the secretion of soluble proteins and EVs is particularly important. Sze et al. (2007) conducted proteomic profiling of secreted products from MSCs, and this revealed that MSCs secrete many membrane vesicles and cytosolic proteins.

Extracellular vesicles are particles that are nanometers in size and enveloped by membranes, and they transfer substances between cells. There are four categories of extracellular vesicles—microvesicles, exosomes, oncosomes, and apoptotic bodies—and this categorization is based on particle size and release mechanism (Zaborowski et al., 2015). The International Society for Extracellular Vesicles also provides a series of additional parameters to distinguish EVs. Due to overlapping size ranges, compositions, and a lack of specific surface markers, we use the term “EVs” in our review instead of specific distinctions unless the specific biogenesis pathway and purity have been determined (Théry et al., 2018).

**FIGURE 2:**
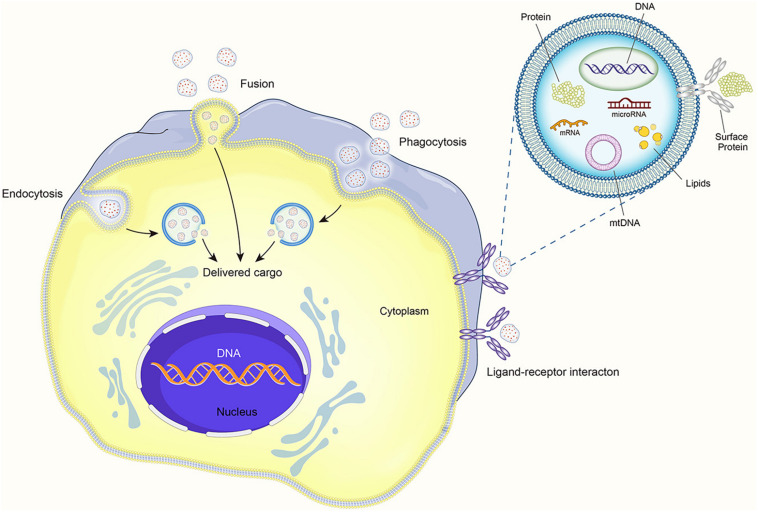
The process of extracellular vesicles communicating with target cells.

It is believed that EVs can produce therapeutic effects through pleiotropic mechanisms. They can directly activate cell surface receptors, integrate with membranes, and transfer transcription factors, proteins, miRNAs, lncRNAs, and mRNAs to receptor cells (Phinney and Pittenger, 2017). Thus, MSCs appear to pack many factors into EVs and distribute them throughout the body *via* the circulatory systromal. The distribution of miRNA in exosomes reveals cellular pathophysiology and can alter various biological processes. Increasing data indicate that EVs and their contents, especially miRNAs, affect the pathophysiology of various diseases, including autoimmune diseases (Mirzaei et al., 2021). The EV contents also regulate many signaling pathways (Bahrami et al., 2021). EVs coat regulatory proteins and RNAs with a phospholipid envelope, which improves the efficiency of intracellular delivery and thereby allows MSCs to rapidly respond to stimuli (Lai et al., 2010). The contact of EVs with recipient cells can occur by three mechanisms: direct interaction with signal receptors on target cells, fusion with the recipient cell plasma membrane and subsequent transfer of cargo, and internalization by the recipient cell (see Figure 2).

### Extracellular Vesicles in Cell-Free Therapies

Most of the published literature on MSCs have sought to recapitulate their properties and functions with the hope of using MSCs in animal disease models. In tissue regeneration, adult MSCs have been used for tissue repair in laboratory and clinical research; however, their proliferation and differentiation capabilities weaken with age. Human pluripotent stem cells have garnered attention from researchers due to their ability to continuously differentiate; however, there are significant concerns about tumorigenicity, genomic instability, and immunogenicity (Nguyen et al., 2021). Sometimes the MSC therapeutics display incompatibility with human blood; the high levels of tissue factor (TF/CD142) could be a key trigger of coagulation (Caplan et al., 2019; Moll et al., 2019). George et al. (2018) detected the TF expression from samples from bone marrow, adipose, amniotic fluid, umbilical cord, multi-potent adult progenitor cell donors, and bone marrow mononuclear cells, and all exhibited evidence of promoting coagulation. At present, although there is no experiment to compare the coagulation promoting ability of MSCs and derived EVs, Berckmans et al. (2019) proved that the EVs detected in the blood of healthy humans promoted fibrinolysis rather than coagulation. Thus, MSC-derived EVs may provide a new therapeutic paradigm for lower-risk cell-free therapies (Phinney and Pittenger, 2017).

Weekly intra-articular injections of human embryonic MSC-derived EVs have been shown to rescue the retardation of osteochondral regeneration in a rat model, and only fibrous repair tissues were found in the control group (Zhang et al., 2016). Taisuke et al. have also shown that EV-free conditioned culture medium failed to repair bone fractures although many cytokines were present, whereas the MSC-derived EVs promoted the healing process. Notably, miR-4532, miR-125b-5p, miR-4516, miR-338-3p, and miR-548aa were expressed at higher levels in the MSC-derived EV group. This suggests that miRNAs are the basis of EVs function in this context (Furuta et al., 2016). Contact between menstrual MSCs and cortical neurons has been shown to inhibit neurite outgrowth; however, EVs have also been shown to promote nerve growth and have great therapeutic potential for treating neurodegenerative pathologies (Lopez-Verrilli et al., 2016). The protective mechanisms of EVs are demonstrated mainly through the activation of proliferative and regenerative responses. In the model liver injury mouse, exosome treatment markedly increased the levels of NF-κB and STAT3, and the interleukin (IL)-6/STAT3 pathway induced cell cycle progression from G1 to S phase as indicated by the increased levels of cyclin D1 and PCNA (Tan et al., 2014). EVs have also been shown to significantly accelerate wound healing. Wnt4 from EVs promotes β-catenin nuclear translocation, and this process quickens re-epithelialization (Zhang et al., 2015). In the treatment of ischemic tissue-related diseases, MSC-derived EVs enhance the formation of vein endothelial cells and inhibit T cell growth and function; this reduces the size of the infarction and restrains the inflammatory response (Teng et al., 2015). Due to the role of EVs in modulating immune responses and restoring tissue homeostasis, EVs have been used to treat osteoarthritis (Zhu et al., 2017), hepatic disease (Lou et al., 2017), and other pathologies. For lung diseases, including chronic obstructive pulmonary disease and lung cancer, EVs have been shown to improve the lung microenvironment and function (Fujita et al., 2015).

## Melatonin Mediates MSC Osteogenesis, Chondrogenesis, and Adipogenesis

Melatonin has been shown to participate in bone metabolism, and 0.01–1 mM melatonin stimulates osteogenesis by upregulating COL-I and OPN expression and secretion (Dalla-Costa et al., 2020). Meanwhile, low dose of melatonin can ameliorate the osteoporosis induced by high-dose glucocorticoids *via* the PI3K/AKT and BMP/Smad signaling pathways (Zhao et al., 2020).

The effects of melatonin on MSCs osteogenesis have been gradually documented. Radio et al. (2006) found that culturing human adult MSCs in osteogenic medium supplemented with melatonin significantly increased osteogenesis. Clathrin-mediated endocytosis, MEK1/2 and ERK1/2, epidermal growth factor receptors, and metalloproteinase were necessary for this increase in osteogenesis, whereas PKA was not (Radio et al., 2006). Melatonin production decreases with age. Work by Paulose et al. (2019) has shown that the melatonin levels in older mice are significantly lower than in younger mice. Decreased melatonin production in the elderly may be a cause of osteoporosis. In one study, researchers successfully used the BMP inhibitor Noggin to block melatonin-induced osteogenesis, which suggests that BMP/ERK/Wnt signaling is the pathway by which melatonin promotes osteogenesis (Park et al., 2011). Within the BMP family, BMP9 is considered one of the most effective in MSCs. By inducing Smad1/5/8 translocation, melatonin effectively enhances the bone formation of MSCs (Jiang et al., 2019). MT2-dependent NF-κB signaling downregulates osteoclastogenesis *via* RANKL paracrine secretion, which is also a potential mechanism by which melatonin affects MSC osteogenesis (Zhou et al., 2020). Certain miRNAs are also associated with MSC osteogenesis—for example, miR-92b-5p, which targets ICAM-1, is highly expressed after melatonin administration and regulates osteogenesis (Li et al., 2019).

Determining the mechanism by which melatonin regulates the osteogenic differentiation of MSCs lays a foundation for cell-free therapies. Nevertheless, some experiments have produced conflicting results. In contrast to previous findings, one study reported that physiological melatonin concentrations (1 pM–10 nM) inhibited the osteogenic differentiation of human periodontal ligament stromal cells, while 1 μM melatonin (the lowest pharmacological concentration) promoted it (Zheng et al., 2020). The acute melatonin treatment was also more effective than chronic exposure, which may be due to the desensitization of the melatonin receptors (Radio et al., 2006). Different concentrations of melatonin and/or different sources of stromal cells may also cause different outcomes among researchers.

Melatonin is also heavily involved in MSCs chondrogenesis, and this effect has been widely documented and applied to treating degenerative cartilage repair. Zhang et al. (2013) first identified MT1 and MT2 melatonin receptors in rat growth plate chondrocytes (GPCs). Wang et al. (2014) isolated and cultured GPCs from adolescent idiopathic scoliosis (AIS) patients, and they found that the MT2 mRNA levels were significantly lower than in healthy controls. They suggested that reduced MT2 expression may suppress the effects of melatonin on MSC chondrogenesis, and this may result in abnormal endochondral ossification in AIS patients. The administration of melatonin also enhances the synthesis of articular chondrocytes *via* the TGF-β signaling pathway (Pei et al., 2009). Because chondrocytes exist in the articular cartilage, melatonin-induced chondrogenic differentiation also has a significant therapeutic effect on osteoarthritis (Hosseinzadeh et al., 2016). Osteoarthritis (OA) is a degenerative joint disease that is primarily caused by reactive oxygen species (ROS). The effective treatment of arthritis involves both the regeneration of cartilage and the inhibition of the inflammatory environment. Sleep has been shown to aid in cartilage repair, and circadian rhythm disruption is considered a risk factor for OA (Chen et al., 2020). Melatonin is therefore useful in this context because it is both a free radical scavenger and an antioxidant, and it can modulate inflammation. The chondroprotective effects of melatonin are correlated with decreased reactive oxygen species, preserved superoxide dismutase, and decreased expression of matrix metalloproteinases (Liu et al., 2014). In RNA sequencing experiments, researchers showed that circRNA3503 is upregulated after melatonin-induced cell sleep. The researchers further constructed circRNA3503-loaded EVs from synovium mesenchymal stromal cells and demonstrated their utility for alleviating OA (Tao et al., 2021).

Because melatonin has been implicated in body weight control, further research is needed to determine whether it can regulate adipogenesis. Melatonin inhibits 3T3-L1 preadipocytes by decreasing the activity of the adipogenic transcription factor C/EBPbeta (Alonso-Vale et al., 2009). In MSCs, researchers have found that melatonin inhibits adipogenesis and promotes osteogenesis by suppressing PPARγ expression and enhancing Runx2 expression. Melatonin also downregulates several markers of terminal adipocyte differentiation, such as lipoprotein lipase, adipocyte protein 2, leptin, and adiponectin (Zhang et al., 2010; Alvarez-García et al., 2012). Further work is needed to explore the different mechanisms by which melatonin regulates adipogenesis and how these might have clinical applications (see Table 1).

**TABLE 1:** Melatonin mediates in mesenchymal stromal cell (MSC) osteogenesis, chondrogenesis, and adipogenesis.

**Cell source**	**Concentration**	**Time point**	**Effect**	**Mechanism**	**References**
Human adult mesenchymal stromal cell	50 nM	Cotreatment	ALP expression↑ Osteogenesis↑	MT2 receptor↑, MEK/ERK (1/2) ↑	Radio et al., 2006
Bone marrow	10 or 100 nM	Cotreatment	Osteogenesis↑ Extracellular mineralization↑	MT2 receptor↑, NF-κB ↑	Zhou et al., 2020
Bone marrow	10 mmol/L	Cotreatment	ALP, collagen-1, BMP2, BMP4, Runx2, Sp7, OCN, and OPN↑	miR-92b-5p↑, ICAM-1↑	Li et al., 2019
Periodontal ligament	1 pM–1 μM	Cotreatment	Osteogenesis ↓ Osteopontin (OPN) and osteocalcin (OCN) ↓	ATP, ROS, NAD+/NADH↓	Zheng et al., 2020
Bone marrow		Cotreatment, slow-release	ALP↑, osteopontin, and osteocalcin↑, calcium deposit ↑	runx2↑	Zhang et al., 2013
Synovium	1 μM	Cotreatment	Rescues the IL-1β and TNF-α; impaired chondrogenesis of MSCs	ROS↓, SOD↑, MMPs↓	Liu et al., 2014
Bone marrow	0, 10^–8^, 10^–6^, and 10^–4^ M	Pretreatment	Adipogenesis↓ osteogenesis ↑	PPARγ↑ Runx2 ↓	Alonso-Vale et al., 2009
Bone marrow	50 nM	Cotreatment	Adipogenic differentiation↓ at the early stage of adipogenic differentiation	ROS↓, phosphorylating ERK/GSK-3β↓	Rhee and Ahn, 2016
Bone marrow	50 nM	Cotreatment	Chondrogenesis↑	MT receptor-dependent pathway↑	Gao et al., 2014
Bone marrow	50 nM	Cotreatment	Pellet size, matrix↑ Chondrogenesis↑ Apoptosis ↓	IL-1β-induced activation of NF-κB signaling↓	Gao et al., 2018

*↑ represents up-regulated, ↓ represents down-regulated.*

## Melatonin Enhances the Anti-Inflammatory Effects of Mesenchymal Stromal Cells and their Derived Extracellular Vesicles

### Melatonin and Mesenchymal Stromal Cells

The inflammatory internal microenvironment weakens the therapeutic efficacy of MSCs *in vivo*, but recent work has shown that melatonin can increase MSC survival and produce a synergistic effect that alleviates inflammation, apoptosis, and oxidative stress (Hu and Li, 2019). Melatonin can act as a highly effective natural antioxidant with multiple mechanisms of action. Oxidative stress refers to a state of imbalance between oxidation and antioxidation, and it is the negative effect of free radicals in the body (Sies, 2015). Melatonin and some of its metabolites, including 6-hydroxymelatonin, cyclic 3-hydroxymelatonin, AFMK, and *N*-acetyl-5-methoxykynuramine, can directly bind to reactive oxygen and reactive nitrogen species. Cyclic 3-hydroxymelatonin is even considered a biomarker for *in vivo* detection of hydroxyl radicals. Furthermore, melatonin can promote the activity of antioxidant enzymes while suppressing pro-oxidant enzymes. Melatonin can also exert its antioxidant functions by chelating transition metals (Carrascal et al., 2018; Hardeland, 2019).

Ischemic injury can be caused by oxidative stress and excessive inflammation, and strategies to prevent ischemic injury should focus on preventing inflammation and eliminating ROS and oxidative stress (Wang et al., 2021). There are various challenges for treating ischemic disease with MSCs, including the low survival rate of MSCs in the hypoxic–ischemic environment. However, combination treatments of melatonin and MSCs have shown great potential for treating ischemic injury and resolving inflammation. Researchers have found that combined melatonin and MSC treatment is superior to melatonin or MSCs alone for treating common ischemic injuries including hind limb ischemia disease (Lee et al., 2017), focal cerebral ischemia disease (Tang et al., 2014), cardiac ischemia disease (Wang et al., 2015), acute lung ischemia–reperfusion injury (Yip et al., 2013), and small bowel ischemia–reperfusion injury (Chang et al., 2015). Certain infectious diseases, including sepsis-induced acute kidney injury (Chen et al., 2014b), sepsis-induced acute lung injury (Chen et al., 2014a), and acute interstitial cystitis (Chen et al., 2014c), can also be ameliorated by treatment with melatonin and MSCs. Tang et al. (2014) proposed that melatonin improves MSCs survival by the activation of the ERK1/2 signaling pathway and that MSCs treated with melatonin have increased vascular endothelial growth factor. Therefore, the melatonin-treated MSCs show greater therapeutic potential for angiogenesis and neurogenesis in focal cerebral ischemia disease (Tang et al., 2014). In the ischemic myocardium model, melatonin protected MSCs from hypoxia/serum deprivation (Hy/SD)-induced cell death *in vitro*, and this was mainly due to the decreased production of intracellular reactive oxygen and upregulation of the BCL2-associated X/B-cell lymphoma-2 ratio. Furthermore, melatonin pretreatment increased the levels of phospho-p38 MAPK and phospho-ERK1/2 in Hy/SD-treated MSCs (Wang et al., 2015).

MSCs senescence is also a barrier to effective therapeutics. In a chronic kidney disease (CKD) model, MSC senescence was accelerated. Melatonin can protect MSCs from senescence by upregulating the cellular prion protein (PrP^c^) expression, which enhances mitochondrial dynamics and metabolism (Han et al., 2019). Interestingly, several researchers have found that apoptotic MSCs are more effective than live MSCs for some purposes and that the contents released from apoptotic MSCs may improve their function *via* endothelial cells (Yip et al., 2013; Weiss and Dahlke, 2019; Liu H. et al., 2020). Phosphatidylserine is exposed on the surface of apoptotic cells and recognized by macrophages and dendritic cells. Macrophages then engulf the apoptotic MSCs and upregulate the secretion of anti-inflammatory cytokines, including IL-10 and TGF-β. Thus, apoptotic MSCs downregulate the innate and adaptive immune systromals, which might result in greater therapeutic effectiveness (Thum et al., 2005) (see Table 1).

### Melatonin and MSC-Derived Extracellular Vesicles

An abundance of literature has confirmed the anti-inflammatory effects of melatonin, and the effects of melatonin on MSCs have been widely explored. In recent years, melatonin and MSC-derived EVs have received increased attention from researchers (see Table 2). Some studies have shown that the anti-inflammatory effects of melatonin occur *via* EVs—for example, in a mouse model of obesity, melatonin was reported to exert its anti-inflammatory effects by increasing the levels of adipose-derived exosomal α-ketoglutarate (αKG). αKG then attenuated STAT3 and NF-κB signaling *via* adipocyte oxoglutarate receptor 1. The same authors also confirmed that the transportation of exosomal αKG to macrophages increased the ratio of M2 macrophages to M1 macrophages (Liu et al., 2018). M1 macrophages are classically activated macrophages that produce pro-inflammatory cytokines, such as IL-1β and TNF-α, and result in an inflammatory response. M2 macrophages, on the other hand, are optionally activated macrophages that produce anti-inflammatory cytokines (Gordon and Martinez, 2010). EVs that transfer miR-34a, miR-124, and miR-135b to target cells can also increase the ratio of M2 to M1 macrophages (Heo et al., 2020). Wei et al. have also found that melatonin treatment of MSCs alters the ratio of M2 to M1 macrophages. They found that the anti-inflammatory effects of melatonin-pretreated MSC-derived EVs occur due to the upregulation of PTEN and the inhibition of AKT phosphorylation; this promotes M2 macrophage polarization and leads to the downregulation of IL-1β, TNF-α, and iNOS. Combined melatonin-EV anti-inflammatory therapy can be applied to a variety of disease scenarios, including diabetic wound healing (Liu W. et al., 2020). For colon dextran sulfate sodium-induced acute colitis, the melatonin–EV treatment significantly reduced inflammation, oxidative stress, apoptosis, and fibrosis to improve clinical symptoms (Chang et al., 2019).

**TABLE 2:** Melatonin enhances the activity of extracellular vesicles (EVs) to rescue the function of multiple organs.

**Disease**	**EVs resource**	**Effect**	**Mechanism**	**References**
Inflammatory state	Adipose-derived MSCs	Anti-inflammatory mRNA expression↑	Modulation *via* exosomal miRNAs miR-34a, miR-124, and miR-135b	Heo et al., 2020
Diabetic wound healing	Bone marrow MSCs	Angiogenesis and collagen synthesis↑	M2/M1↑, PTEN↑, phosphorylation of AKT↓	Liu W. et al., 2020
Acute colitis	Adipose-derived MSCs	Colon injury score, expression of inflammatory and DNA-damage markers, and bloody stool improved	NOX-1, NOX-2, MMP-9, NF-κB, iNOS, ICAM-1, and COX-2↓	Chang et al., 2019
Liver ischemia–reperfusion injury	Adipose-derived MSCs	Protect the liver against ischemia-reperfusion injury.	Inhibit the inflammatory, oxidative stress, apoptosis, DNA damage and mitochondrial damage	Sun et al., 2017
Renal ischemia–reperfusion injury	Bone marrow MSCs	Renal damage↓	Oxidative stress, apoptosis, inflammation related genes↓, angiogenesis related genes↑	Alzahrani, 2019
Renal ischemia–reperfusion injury	Bone marrow MSCs	Lower histopathological score of kidney injury	Bax/Bcl2↓	Zahran et al., 2020
Chronic kidney disease	Human adipose tissue-derived MSCs	Protected mitochondrial function, cellular senescence, and proliferative potential of CKD-MSCs, angiogenesis↑	miR-4516↑, cellular prion protein↑	Yoon et al., 2020
Vascular calcification and ageing	Vascular smooth muscle cells/calcifying vascular smooth muscle cells	Attenuated the osteogenic differentiation and senescence of VSMCs or CVSMCs	miR-204/miR-211 in EVs targeted; BMP2 mediated the effect	Xu et al., 2020
*In vitro* Aβ toxicity model	SH-SY5Y cell line	Melatonin inhibited the release of EVs and decrease the amyloid beta load and toxicity by inhibiting exosome release		Ozansoy et al., 2020
Cerebral ischemia-induced pyroptosis	Plasma	Decreased the infarct volume and improved recovery of function	TLR4/NF-κB↓, miRNAs mediated	Wang et al., 2020

*↑ represents up-regulated, ↓ represents down-regulated.*

Ischemia reperfusion injury refers to partial or complete acute artery obstruction. Reperfusion occurs after a period, although the damaged tissue will show a progressive pathology (Binder et al., 2015). For *in vitro* studies of liver ischemia–reperfusion injury, the groups treated with EVs and melatonin had the greatest suppression of oxidative stress, inflammation, and pro-apoptotic factors. For *in vivo* studies, EV and melatonin treatment produced the lowest liver injury scores and plasma aspartate aminotransferase concentrations. The effect of melatonin and EVs was better than that of melatonin alone and even better than that of melatonin and MSCs (Sun et al., 2017). These findings can also be extended to renal ischemia–reperfusion injuries (Alzahrani, 2019; Zahran et al., 2020). Therefore, regimens combining stromal cell-derived EVs and melatonin can effectively treat tissue ischemic injuries, and the mechanism for this phenomenon requires further study.

Chronic kidney disease occurs when the kidneys cannot exert normal function, and this significantly lowers the effectiveness of autologous mesenchymal stromal and stromal cell-based therapies. To address this, Yeo et al. treated CKD-MSCs with melatonin-treated MSC-derived EVs and found that the PrP^c^ levels increased, and this was mediated by miR-4516. Treatment with MSC-derived EVs also increased mitochondrial function, cellular aging, and proliferative potential. Thus, EVs treated by melatonin might be a powerful therapeutics strategy in CKD (Yoon et al., 2020).

In Alzheimer’s disease (AD), melatonin was shown to inhibit EVs that spread disease-associated toxic proteins and to prevent the progression of AD (Ozansoy et al., 2020). EVs from plasma and adipocytes have also been shown to reduce inflammation. This could occur because melatonin increases the αKG levels in adipose tissue, and αKG is then transferred to macrophages where it promotes M2 polarization and attenuates STAT3 and NF-κB signaling to reduce inflammation. When plasma-derived EVs were used to treat cerebral ischemia-induced pyroptosis, they altered signaling through the TLR4/NF-κB pathway (Wang et al., 2020).

## Discussion

An indole hormone primarily secreted from the pineal gland, melatonin has myriad biological functions, including but not limited to endocrine rhythm regulation and anti-inflammation, anti-tumor, and anti-aging functions. Decreased melatonin levels in the elderly are associated with chronic illnesses and insomnia (Gallucci et al., 2014). Thus, elderly individuals may benefit from taking melatonin supplements or consuming foods rich in melatonin, and this may help to recover pineal function and prevent chronic diseases. This hypothesis should be evaluated in clinical trials.

In recent decades, many studies have explored the regulatory roles of melatonin on MSCs and their derived EVs. At present, there is compelling evidence that melatonin can promote MSCs osteogenesis and chondrogenesis and inhibit adipogenesis, although it is essential to determine the optimal concentration and administration time. These findings lay a foundation for adding melatonin to MSCs induction media. However, the mechanisms for the effects of melatonin on MSCs need to be further clarified to avoid the occurrence of side effects and unexpected complications. Melatonin also inhibits the release of inflammatory factors, which promotes the proliferation of MSCs, reduces aging and death of MSCs, and improves function. Furthermore, melatonin can regulate ROS levels and promote the expression of antioxidant genes, thereby producing a protective and anti-apoptotic effect on MSCs.

As MSCs research has advanced, there has been a greater appreciation for the importance of paracrine signaling in MSC function, and EVs are critical for paracrine signaling. MSC-derived EVs are crucial for cell-to-cell communication, and they regulate cell fate and can play therapeutic roles. MSC-derived EVs contain cargos of proteins, DNA, lipids, cytokines and growth factors, mRNAs, and regulatory miRNAs. Compared to traditional MSC-based cell therapy approaches, EV-based cell-free therapy can potentially overcome the negative side effects of transplanted cells, such as immune rejection. Therefore, there is great interest in replacing MSC-based cell therapies with MSC-derived EV therapies. In the present review, we also highlighted the application of melatonin-treated EVs to various disease models. Generally, the therapeutic effects of melatonin combined with EVs are better than those of melatonin or stromal cells alone. This indicates that EV cell-free therapy has exciting prospects. Further research is needed to apply MSC-derived exosome therapy in tissue engineering and to more clearly elucidate the mechanism(s) of action. There also remains an urgent need to discover new and efficient methods for identifying and extracting EVs. In conclusion, this article reviewed the therapeutic effects of melatonin on mesenchymal stromal cells and their derived EVs and laid a foundation for future cell-free therapy approaches.

## Author Contributions

S-DY and TW: conceptualization. Z-YF: original manuscript preparation. SG: draft correction, supervision, and editing. All authors listed have made a substantial contribution to the manuscript, which is acknowledged and confirmed as the final version of the manuscript.

## Conflict of Interest

The authors declare that the research was conducted in the absence of any commercial or financial relationships that could be construed as a potential conflict of interest.

## Publisher’s Note

All claims expressed in this article are solely those of the authors and do not necessarily represent those of their affiliated organizations, or those of the publisher, the editors and the reviewers. Any product that may be evaluated in this article, or claim that may be made by its manufacturer, is not guaranteed or endorsed by the publisher.
